# The prognostic impact of tumor mutational burden (TMB) in the first-line management of advanced non-oncogene addicted non-small-cell lung cancer (NSCLC): a systematic review and meta-analysis of randomized controlled trials

**DOI:** 10.1016/j.esmoop.2021.100124

**Published:** 2021-04-30

**Authors:** A. Galvano, V. Gristina, U. Malapelle, P. Pisapia, F. Pepe, N. Barraco, M. Castiglia, A. Perez, C. Rolfo, G. Troncone, A. Russo, V. Bazan

**Affiliations:** 1 Department of Surgical, Oncological and Oral Sciences, University of Palermo, Palermo, Italy; 2 Department of Public Health, University Federico II of Naples, Naples, Italy; 3 Marlene and Stewart Greenebaum Comprehensive Cancer Center, University of Maryland School of Medicine, Baltimore, USA; 4 Department of Experimental Biomedicine and Clinical Neurosciences, University of Palermo, Palermo, Italy

**Keywords:** NSCLC, TMB, ICIs, prognostic, meta-analysis

## Abstract

**Background:**

The role of tumor mutational burden (TMB) is still debated for selecting advanced non-oncogene addicted non-small-cell lung cancer (NSCLC) patients who might benefit from immune checkpoint inhibitors (ICIs). Of note, TMB failed to predict a benefit in overall survival (OS) among such patients.

**Materials and methods:**

The purpose of this meta-analysis was to compare efficacy outcomes among first-line immune-oncology (IO) agents versus standard platinum-based chemotherapy (CT) within two subgroups (TMB-low and TMB-high on either tissue or blood). We collected hazard ratios (HRs) to evaluate the association for progression-free survival (PFS) and OS, with the relative 95% confidence intervals (CIs). Risk ratios (RRs) were used as an association measure for objective response rate (ORR).

**Results:**

Eight different cohorts of five randomized controlled phase III studies (3848 patients) were analyzed. In TMB-high patients, IO agents were associated with improved ORR (RRs 1.37, 95% CI 1.13-1.66), PFS (HR 0.69, 95% CI 0.61-0.79) and OS (HR 0.67, 95% CI 0.59-0.77) when compared with CT, thus suggesting a possible predictive role of high TMB for IO regimens. In TMB-low patients, the IO strategy did not lead to any significant benefit in survival and activity, whereas the pooled results of both ORR and PFS were intriguingly associated with a statistical significance in favor of CT.

**Conclusions:**

This meta-analysis resulted in a proven benefit in OS in favor of IO agents in the TMB-high population. Although more prospective data are warranted, we postulated the hypothesis that monitoring TMB, in addition to the existing programmed death-ligand 1 (PD-L1) expression level, could represent the preferable option for future clinical research in the first-line management of advanced non-oncogene addicted NSCLC patients.

## Highlights

• A total of eight cohorts from five RCTs comparing IO and CT were included in this meta-analysis.
• In the TMB-high subgroup, pooled results confirmed the superiority of ICIs over CT in terms of response and survival.
• In addition to PD-L1, monitoring TMB could represent the preferable option for future research in this setting.

## Introduction

Immune checkpoint inhibitors (ICIs), targeting the programmed cell death protein 1 (PD-1)/programmed death-ligand 1 (PD-L1) axis and cytotoxic T-cell lymphocyte antigen 4(CTLA-4) pathway either as single or combination treatments, have recently shown to achieve long-term survival rates in advanced non-oncogene addicted non-small-cell lung cancer (NSCLC) patients.^1^, ^2^, ^3^, ^4^, ^5^ However, despite the evidence of higher efficacy of immune-oncology (IO) treatment strategies compared with standard first-line platinum-based chemotherapy (CT), only a subset of patients seemed to respond.^6^^,^^7^ Hence, in this setting, the discovery of definitive predictive biomarkers for ICI treatment efficacy has become the major issue.^8^ PD-L1 expression and tumor mutational burden (TMB) have been deeply investigated as ICIs predictive biomarkers in different randomized trials.^9^, ^10^, ^11^ However, despite the approval of PD-L1 expression as a standard biomarker for ICIs in the management of advanced NSCLC patients,^1^^,^^7^ response prediction was revealed to be imperfect with a not-negligible percentage of patients experiencing resistance either at baseline or during treatment.^12^ Similarly, tissue-based TMB turned out to be a promising predictive biomarker as reported by Carbone et al. in the subgroup analysis of the CheckMate-026 trial of first-line PD-1 inhibitor single treatment.^13^ In the era of precision oncology, careful attention should be paid to the emerging role of TMB estimated by circulating tumor DNA in blood, a minimally invasive approach that could similarly predict survival in patients receiving ICIs showing good correlation with tissue TMB.^14^ Nonetheless, the clinical utility of TMB as a biomarker together with PD-L1 evaluation remains an enigma further complicated by the variety of measuring assays and algorithms.^15^, ^16^, ^17^, ^18^, ^19^ Interestingly, no significant association between PD-L1 expression and TMB has been observed,^20^ even when comparing the results obtained from the same NSCLC tissue samples.^21^^,^^22^

As far as TMB is concerned, discordant results have emerged when progression-free survival (PFS) and overall survival (OS) were considered.^23^, ^24^, ^25^, ^26^ As highlighted in CheckMate clinical trials, despite an initial improvement in PFS in advanced NSCLC patients with high TMB treated with dual ICI combination, TMB has been associated with similar OS rates regardless of its level.^10^^,^^11^ Exploratory analyses from different first-line KEYNOTE randomized trials showed a consistent improvement in major clinical outcomes in the TMB-high population treated with IO monotherapy,^27^^,^^28^ whereas no significant associations emerged between TMB expression and efficacy of ICIs plus chemotherapy in the first-line setting.^29^

Based on the most updated clinical evidence, the purpose of this meta-analysis was to compare objective response rate (ORR), PFS, and OS among first-line IO treatment strategies (single-agent and combination ICIs) versus CT alone within two subgroups (TMB-low and TMB-high in either tissue or blood). Therefore, here we aimed to investigate the association between TMB and NSCLC with the goal of elucidating the clinical and survival impact of TMB in the first-line treatment of advanced non-oncogene addicted NSCLC patients.

## Materials and methods

### Search strategy and study selection

We looked for the results of randomized phase II and III trials comparing patients with histological diagnosis of unresectable or advanced non-oncogene addicted NSCLC [IIIB/IIIC-IV according to the 8th TNM (tumor–node–metastasis) classification and clinical staging system^15^] with high- or low-TMB value in tissue and blood samples. We only included TMB-selected patients receiving IO agents or CT alone in the first-line setting of advanced non-oncogene addicted NSCLC, excluding randomized trials evaluating the association of IO agents with CT to obtain a homogeneous population and avoid sources of bias. We excluded non-randomized or cross-sectional studies, cohort, case-control, and retrospective studies. We also excluded reviews (systematic or not) and meta-analyses. Furthermore, we excluded trials not containing data with at least one outcome of interest, trials with a small sample size (less than 10 patients), and ongoing studies. Studies have been included if patients have undergone IO regimens containing anti-PD-1 (nivolumab or pembrolizumab) or anti-PD-L1 (atezolizumab or durvalumab) agents in association or not with anti-CTLA-4 (ipilimumab or tremelimumab) agents compared with standard platinum-based CT including cisplatin or carboplatin in association with gemcitabine or paclitaxel or pemetrexed, or nab-paclitaxel, according to NSCLC histology (adenocarcinoma, squamous, large cell carcinoma or not otherwise specified). The research was carried out using specific mesh terms such as ‘NSCLC’ and free text terms such as ‘tumor mutational burden’ or ‘TMB’ and ‘survival’ using Boolean operators (Supplementary Figure S1, available at https://doi.org/10.1016/j.esmoop.2021.100124). Data collected on Medline (PubMed), Scopus, and Cochrane-Library database were collected until 30 September 2020, restricting the search to English-only articles; potential abstracts published on the databases of the American Society of Clinical Oncology (ASCO) and European Society of Medical Oncology (ESMO) were also retrieved, as well as results from not-yet-published ongoing studies available on the National Institute of Health (NIH) website (www.clinicaltrials.gov) because they were considered as a source of gray literature. The search protocol was previously registered on the PROSPERO 2020 database: CRD42020179759. The selected outcomes were ORR, defined as the proportion of patients with reduced disease burden (partial response + complete response according to RECIST version 1.1 criteria); PFS, defined as the time interval from randomization to disease progression or death; OS, defined as the time interval between randomization and death from any cause. The data collected for these outcomes were stratified according to the high- or low-TMB value. The threshold to determine the high- or low-TMB value was defined by the authors of the selected studies, except for the atezolizumab trial (IMpower110), considering that a statistical and clinical benefit has been observed according to a prespecified TMB cut-off value of ≥16 mutations/megabase.^30^ Only data from studies that enrolled patients aged ≥18 years with no sex restrictions were collected. The initial selection of trials was carried out independently by two authors (A.G. and V.G.) who screened and identified the articles considering the previously established inclusion and exclusion criteria. The selected trials were subsequently evaluated for relevant outcomes and included in the final analysis. Any disagreements were discussed and resolved with a third author (A.R.).

### Data extraction and assessment of the quality of the included studies

The data were extracted independently by two authors (A.G. and V.G.) and the disagreements were discussed and resolved with a third author contribution (A.R.). Data were collected in a predefined file in which we reported the trial name, drug protocol, sample size, TMB threshold and source (tissue and blood), the method of detection [whole-exome sequencing (WES) or next-generation sequencing (NGS)] and the results of the selected outcomes (ORR, PFS and OS) stratified according to the high- or low-TMB value. If articles with different follow-up published over time were identified, the most recent article and methodologically effective was selected. Namely, eight different cohorts of five randomized controlled phase III studies (KEYNOTE-042,^28^ CheckMate-227,^10^^,^^31^ CheckMate-026,^13^ MYSTIC^32^ and IMpower110^33^) have been included in the final analysis.

### Statistical analysis

Statistical analysis was carried out using RevMan version 5.3,^34^ and Comprehensive Meta-analysis version 2.2.064.^35^ As already described, the outcomes selected to perform meta-analysis comparisons were ORR, PFS and OS stratified according to the high- or low-TMB value. We collected hazard ratios (HR) to evaluate the association with PFS and OS, with the relative 95% confidence intervals (CI). Risk ratios (RRs) were used as an association measure for ORR, considered as the ratio between the total number of events (in this case, the anticancer responses) on the total number of patients randomized in each group (experimental and control). This study was divided into two phases named standard meta-analysis comparisons and indirect comparisons. We used the standard meta-analytical technique to compare the performance of IO agents versus CT, according to TMB-high or -low for each selected outcome (ORR, PFS, and OS), calculating the logarithm of the HR (logHRs) or the RR (logRR) and their standard error (logSE) for all the randomized clinical trials (RCTs) included in the analysis. A pre-planned subgroup analysis between single-agent ICI (Mono IO) and combination ICIs (Combo IO) versus CT alone was carried out to obtain each subset pooled results, according to low or high TMB. Heterogeneity between studies was assessed using the Cochrane Q test and the inconsistency test (I-squared). In particular, if I-squared was >50%, corresponding to a high risk of heterogeneity, then the meta-analysis was calculated using the random-effect-based model as established by DerSimonian and Laird; otherwise, the meta-analysis was carried out using the fixed-effect-base model according to Mantel-Haenszel.^36^ So, if the HR value is <1, the intervention arm performs better than control. Together, if the RR value is <1, the intervention arm performs worse than the control arm. As regards other potential sources of bias, the possibility of publication bias was explored using Egger's test and producing the related funnel plot for asymmetry. The manuscript was drafted following the Preferred Reporting Items for Systematic Reviews and Meta-Analyzes (PRISMA) guidelines (Supplementary Figure S2, available at https://doi.org/10.1016/j.esmoop.2021.100124). The *P* values were considered significant if *P* ≤ 0.05.

## Results

### Selected studies

**Figure 1:**
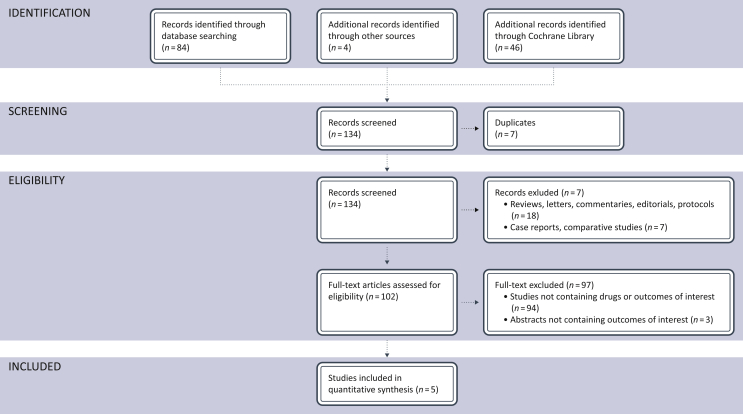
Flow diagram showing the selection algorithm of retrieved papers according to the inclusion/exclusion criteria.

The literature search identified a total of 134 records; 7 records were excluded because of duplicates; 25 records were excluded because of retrospective case reports or non-randomized phase I/II studies, comparative studies, reviews. A total of 102 trials were assessed for eligibility and 97 were excluded because no drugs of interest or data about the principal outcomes of our indirect comparison (ORR, PFS, OS) were reported. Finally, a total of five studies (comprising 3848 patients with available TMB data within eight different cohorts) met our inclusion/exclusion criteria and were included in the final meta-analysis (Figure 1).

### Study characteristics

The baseline characteristics and the outcomes measures of each included trial are reported in Tables 1 and 2, respectively. Quality analysis was carried out to evaluate the overall risk of bias of the randomized trials included in this meta-analysis using the Jadad score, as suggested by the Cochrane Handbook for Systematic Reviews of Interventions.^37^ Briefly, it consists of six domains and those analyzed were the following: allocation concealment; blinding of participants, personnel, and outcome assessors; incomplete outcome data; sequence generation; selective outcome reporting. For each study, the presence of a high risk of bias was defined as ‘High’, whereas the absence of a significant risk of bias was defined as ‘Low’; studies in which the presence of a risk of bias was difficult to quantify were defined as ‘Unclear’. Specifically, two different authors (A.G. and V.G.) assessed the risk of selective outcome reporting bias and any uncertainties were resolved by consensus.

**Table 1:** The main baseline characteristics of each included trial considered in this meta-analysis

Study	TMB-evaluable patients, *n* (%)	Treatment arm	Number of patients	Detection method	Threshold defined	Sample
KEYNOTE-042^28^	793/1274 (62.2)	Pembrolizumab versus CT	180 versus 165 (TMB-high); 234 versus 214 (TMB-low)	WES	175 mut/exome	Tissue
CheckMate 227 part 1^31^-2^10^	679/1166 (58.2)	Nivolumab + ipilimumab versus CT	139 versus 160 (TMB-high); 191 versus 189 (TMB-low)	NGS (FoundationOne®CDx)	10 mut/mb	Tissue
CheckMate 026^13^	312/541 (57.6)	Nivolumab versus CT	47 versus 60 (TMB-high); 111 versus 94 (TMB-low)	WES	243 missense mut	Tissue
MYSTIC^32^	315/744 (42.3)	Durvalumab + tremelimumab versus CT	60 versus 67 (TMB-high); 104 versus 84 (TMB-low)	NGS (FoundationOne®CDx)	10 mut/Mb	Tissue
MYSTIC^32^	296/746 (39.6)	Durvalumab versus CT	60 versus 67 (TMB-high); 85 versus 84 (TMB-low)	NGS (FoundationOne®CDx)	10 mut/Mb	Tissue
MYSTIC^32^	523/744 (70.2)	Durvalumab + tremelimumab versus CT	64 versus 70 (TMB-high); 204 versus 185 (TMB-low)	NGS (Guardant OMNI®)	20 mut/Mb	Blood
MYSTIC^32^	541/746 (72.5)	Durvalumab versus CT	77 versus 70 (TMB-high); 209 versus 185 (TMB-low)	NGS (Guardant OMNI®)	20 mut/Mb	Blood
IMpower110^33^	389/554 (70.2)	Atezolizumab versus CT	87 (TMB-high); 302 (TMB-low)	NGS (FoundationOne®CDx)	16 mut/Mb	Blood

CT, platinum-based chemotherapy; Mb, megabase; mut, mutations; NGS, next-generation sequencing; TMB, tumor mutational burden; WES, whole-exome sequencing.

**Table 2:** Clinical outcomes measures stratified according to tissue TMB status

Study	ORR (TMB-high) *n* (%)	ORR (TMB-low) *n* (%)	PFS (TMB-high) HR (95% CI)	PFS (TMB-low) HR (95% CI)	OS (TMB-high) HR (95% CI)	OS (TMB-low) HR (95% CI)
KEYNOTE-042^28^	62/180 (34.4) versus 51/165 (30.9)	44/234 (18.8) versus 48/214 (22.4)	0.75 (0.59-0.95)	1.27 (1.04-1.55)	0.62 (0.48-0.80)	1.09 (0.88-1.36)
CheckMate 227 part 1^31^-2^10^	63/139 (45.3) versus 43/160 (26.9)	N.A.	0.58 (0.43-0.77)	1.07 (0.84-1.35)	0.68 (0.51-0.91)	0.75 (0.59-0.94)
CheckMate 026^13^	N.A.	N.A.	0.62 (0.38-1.00)	1.82 (1.30-2.55)	1.1 (0.64-1.88)	0.99 (0.71-1.4)
MYSTIC^32^ tissue D + T versus CT	N.A.	N.A.	0.97 (0.63-1.49)	1.98 (1.42-2.78)	0.72 (0.48-1-09)	1.39 (1.00-1.92)
MYSTIC^32^ tissue D versus CT	N.A.	N.A.	0.86 (0.55-1.33)	1.49 (1.95-2-13)	0.70 (0.47-1.06)	1.26 (0.90-1.77)
MYSTIC^32^ blood D +	31/64 (48.4) versus 15/70 (21.4)	34/204 (16.7) versus 58/185 (31.4)	0.53 (0.34-0.81)	1.55 (1.23-1.94)	0.49 (0.32-0.74)	1.16 (0.93-1.45)
MYSTIC^32^ blood D versus CT	23/77 (29.9) versus 15/70 (21.4)	43/209 (20.6) versus 58/185 (31.4)	0.77 (0.52-1.13)	1.19 (0.94-1.50)	0.72 (0.50-1.05)	0.93 (0.74-1.16)
IMpower110^33^	N.A.	N.A.	0.55 (0.33-0.92)	1.00 (0.78-1.29)	0.75 (0.41-1.35)	1.07 (0.77-1.47)

CI, confidence interval; CT, platinum-based chemotherapy; D, durvalumab; HR, hazard ratio; N.A., not available; ORR, overall response rate; OS, overall survival; PFS, progression-free survival; T, tremelimumab; TMB, tumor mutational burden.

### Meta-analysis results

Eight cohorts of five RCTs for a total of 3848 patients with available TMB data evaluated first-line IO regimens containing anti-PD-1 (nivolumab or pembrolizumab) or anti-PD-L1 (atezolizumab or durvalumab) agents in association or not with anti-CTLA-4 (ipilimumab or tremelimumab) agents compared with standard platinum-based CT in unresectable or advanced non-oncogene-addicted NSCLC. Moreover, we evaluated the specific contribution of single-agent and combination ICIs (Mono IO and Combo IO, respectively) when directly compared with CT alone.

#### IO agents versus platinum-based CT – high TMB

**Figure 2:**
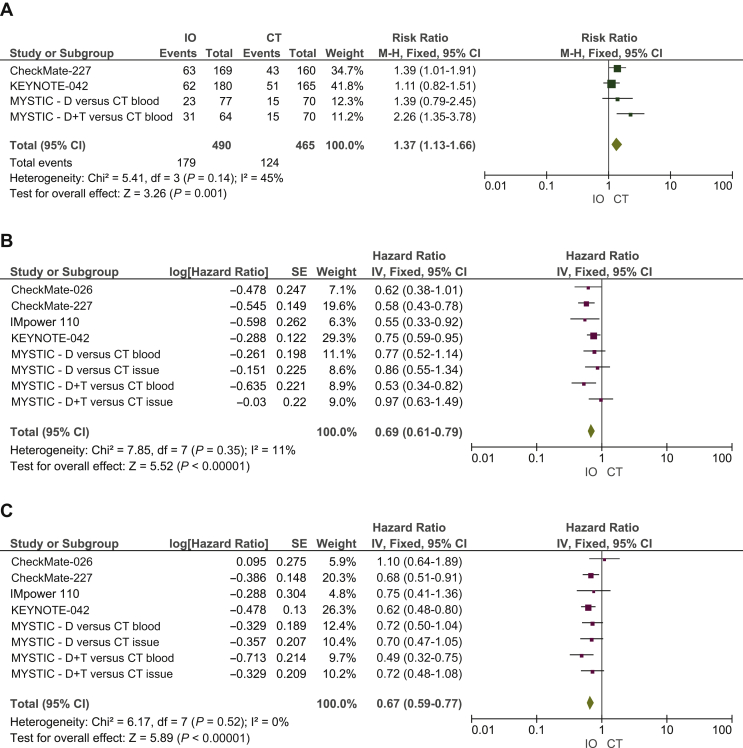
Meta-analysis results including forest plots of (A) RR of ORR, (B) HR of PFS and (C) OS in patients with high TMB assigned to receive first-line IO regimens versus CT. CI, confidence interval; CT, platinum-based chemotherapy; D, durvalumab; df, degree freedom; HR, hazard ratio; IO, immuno-oncology; IV, inverse variance; M-H, Mantel-Haenszel; ORR, objective response rate; OS, overall survival; PFS, progression-free survival; RR, risk ratio; SE, standard error; T, tremelimumab; TMB, tumor mutational burden.

Specifically, 1373 patients were TMB-high. In this setting, our pooled results showed clear statistically significant differences in terms of ORR (RR 1.37, 95% CI 1.13-1.66), PFS (HR 0.69, 95% CI 0.61-0.79) and OS (HR 0.67, 95% CI 0.59-0.77), favoring IO regimens when compared with standard CT (Figure 2).

#### IO agents versus platinum-based CT – low TMB

**Figure 3:**
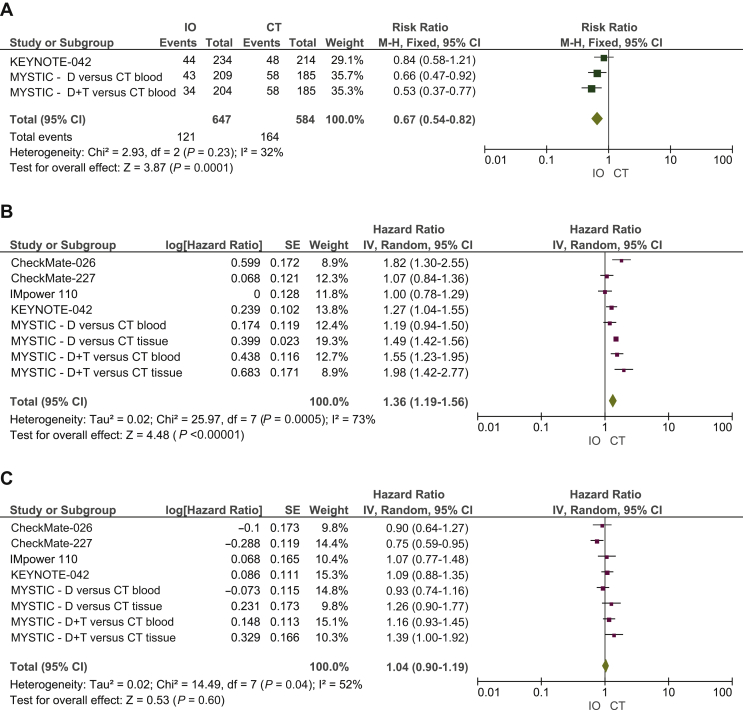
Meta-analysis results including forest plots of (A) RR of ORR, (B) HR of PFS and (C) OS in patients with low TMB assigned to receive first-line IO regimens versus CT. CI, confidence interval; CT, platinum-based chemotherapy; D, durvalumab; df, degree freedom; HR, hazard ratio; IO, immuno-oncology; IV, inverse variance; M-H, Mantel-Haenszel; ORR, objective response rate; OS, overall survival; PFS, progression-free survival; RR, risk ratio; SE, standard error; T, tremelimumab; TMB, tumor mutational burden.

A total of 2475 patients were TMB-low. When directly comparing ICIs versus CT alone in this subgroup, IO regimens did not produce advantages in terms of ORR (RR 0.67, 95% CI 0.54-0.82), with no significant benefit in PFS (HR 1.36, 95% CI 1.19-1.56). Neither IO nor CT resulted in an OS benefit (HR 1.04, 95% CI 0.90-1.19) (Figure 3).

#### Single-agent ICI (Mono IO) versus platinum-based CT – high TMB

As far as ORR is concerned, the administration of single-agent IO regimens reflected a trend favoring disease debulking, albeit not statistically significant (RR 1.18, 95% CI 0.90-1.54). In our analysis, this effect also turned into a clear effect in PFS (HR 0.73, 95% CI 0.62-0.86) and OS (HR 0.70, 95% CI 0.59-0.83), supporting the use of single-agent ICIs in the TMB-high subgroup (Supplementary Figure S3, available at https://doi.org/10.1016/j.esmoop.2021.100124).

#### Single-agent ICI (Mono IO) versus platinum-based CT – low TMB

Even if only two cohorts could be considered when evaluating ORR in this setting, the administration of CT alone produced a greater result over single-agent IO (RR 0.74, 95% CI 0.58-0.95), however with no significant benefit in OS (HR 1.03, 95% CI 0.91-1.17). Interestingly, PFS pooled results underlined a statistical significance in favor of standard CT over IO regimens (HR 1.32, 95% CI 1.12-1.56) in TMB-low patients (Supplementary Figure S4, available at https://doi.org/10.1016/j.esmoop.2021.100124).

#### Combination ICIs (Combo IO) versus platinum-based CT – high TMB

Our pooled results showed clear statistically significant differences in terms of all the outcomes including ORR (RR 1.70, 95% CI 1.06-2.72), PFS (HR 0.66, 95% CI 0.47-0.92), and OS (HR 0.64, 95% CI 0.52-0.78), favoring the combination ICI strategy over standard CT in TMB-high patients (Supplementary Figure S5, available at https://doi.org/10.1016/j.esmoop.2021.100124).

#### Combination ICIs (Combo IO) versus platinum-based CT – low TMB

As regards ORR, even if only data of the blood cohort from the MYSTIC trial could be evaluated, the administration of CT alone was more active in terms of response (RR 0.53, 95% CI 0.37-0.77), however with no significant difference in OS (HR 1.05, 95% CI 0.74-1.50). Intriguingly, PFS pooled results demonstrated a great benefit in favor of CT alone over the combination strategy (HR 1.46, 95% CI 1.05-2.05) in TMB-low patients (Supplementary Figure S6, available at https://doi.org/10.1016/j.esmoop.2021.100124).

### Risk of bias assessment

In our analysis, publication bias Egger's test was calculated for every outcome showing no statistical significance (Supplementary Figure S7, available at https://doi.org/10.1016/j.esmoop.2021.100124). The overall quality assessment was evaluated according to the CONSORT checklist statement. We reported an average good quality of all trials. Some problems related to ‘Blinding of participants and personnel’ (performance bias) domains because studies were reported as open-label designs (Supplementary Figure S8, available at https://doi.org/10.1016/j.esmoop.2021.100124).

## Discussion

This meta-analysis focused the attention on the clinical utility of TMB as a predictive biomarker of the response to immunotherapy in the first-line setting of advanced non-oncogene addicted NSCLC patients treated with ICIs compared with standard CT alone. Most selected studies evaluating the potential role of TMB in such patients seemed to suggest that TMB would not be ready for prime-time yet, requiring a careful assessment for its routine use in clinical practice.^38^, ^39^, ^40^, ^41^ Accordingly, there is still the need to identify those TMB-high patients who do not respond to immune checkpoint blockade, aiming to evaluate additional genetic features associated with the anticancer immune response.^42^^,^^43^ Furthermore, whether all the different IO agents are equally effective across the TMB spectrum in advanced-stage NSCLC patients is still unknown.^44^, ^45^, ^46^ In this context, the lack of standardization for TMB calculation with different platforms reporting different TMB expression thresholds must be considered.^47^, ^48^, ^49^

Of note, recent analyses in advanced NSCLC patients who received ICIs highlighted that TMB and PD-L1 expression may be uncorrelated, suggesting that they could be independent predictive biomarkers that can each contribute to the identification of patients for immune-based therapy.^50^, ^51^, ^52^ In this scenario, we hypothesized that PD-L1 might not be the sole guiding biomarker in the first-line setting of advanced NSCLC without targetable genetic alterations and inferred that the complementary use of tissue TMB monitoring could be implemented in the clinic to improve patient selection. Since direct comparison studies will be unlikely in this setting, we compared different IO agents versus standard CT alone to identify any potential differences in efficacy outcomes within two main TMB subgroups. We encompassed publicly available results from first-line randomized phase III studies testing IO strategies, including direct randomized evidence on treatments of interest along with indirect evidence and exploratory analyses from randomized studies with platinum CT as the common comparator. Even if the trial designs slightly differed according to the different available ICI combinations, the setting and the efficacy outcomes stratified by TMB status were the same in all these studies. Overall, 35% and 65% of the patients included in this meta-analysis were TMB-high and -low, respectively.

The results of this meta-analysis demonstrated that, when assigned to receive IO agents versus platinum-based CT, TMB-high patients showed a remarkable effect in ORR and a statistically significant benefit in PFS along with an unprecedented OS improvement compared with those patients featuring a low-TMB status. Conversely, in TMB-low patients, no OS benefit in favor of one treatment or another has been observed, whereas CT was more active and associated with a reduced risk of progressing disease when compared with IO agents. Focusing on the pre-planned subgroup analysis, our results confirmed that, when compared with CT alone, both single-agent and combination ICIs were associated with a statistically significant advantage in terms of PFS and OS within the TMB-high subgroup; however, as regards activity in this setting, the dual checkpoint blockade appeared to be associated with an increased response rate whereas the IO monotherapy did not reach the statistical significance in the subgroup of patients with high TMB. As far as the TMB-low subgroup is concerned, the IO strategy did not lead to any significant benefit in OS with no advantage in terms of activity and PFS, whereas in this setting, the pooled results of both ORR and PFS were intriguingly associated with a statistical significance in favor of CT over both single-agent and combination ICIs.

Thus, these results demonstrated a significant improvement in efficacy outcomes in TMB-high patients undergoing first-line IO regimens as compared with CT in terms of death risk reduction and higher chances of disease debulking, especially with the administration of combination ICIs. Besides, a possible predictive role of high TMB for IO regimens could be inferred since in the TMB-low subgroup, a statistical significance in activity and progression risk reduction toward CT when compared with IO strategies has been observed, albeit not translating into significative OS differences. Accordingly, despite inconsistencies in definitions and reporting, TMB may act as a predictive biomarker in addition to PD-L1 expression for the selection of the most appropriate patients treated with ICIs. Of note, the greater magnitude of benefit in the TMB-high subgroup appeared to come from two trials (namely, KEYNOTE-042 and CheckMate-227), thus reflecting a higher heterogeneity value in the TMB-low subgroup, although the significant differences in effect size direction affecting the included studies must be considered. These results altogether suggest that TMB would strongly complement PD-L1 expression assessment and may eventually help physicians rule in the most personalized therapeutic approach based on single-agent or combination ICIs in the first-line management of advanced non-oncogene-addicted NSCLC. However, there is a need for further prospective studies using tissue and blood TMB to investigate the prediction accuracy of ICI response and the concordance across testing platforms along with sample quality control and appropriate data interpretation to reliably implement TMB as a viable biomarker in clinical practice.

### Limitations

The present study considered the latest NSCLC results to investigate different performances between first-line IO agents and CT among TMB-selected populations.^53^ Nevertheless, this study had several limitations. First, these results should always be interpreted with caution since meta-analysis is not carried out at the individual-patient level. Secondly, a caveat when interpreting results from this study is the variability of techniques that were used in individual trials for measuring TMB expression levels; in our meta-analysis, this was of no consequence for the overall analysis, but the use of different reporting detection methods and cut-off points would represent an important limitation since the subgroup breakdowns reported in each study were based on differently approved techniques and different gene panel platforms; namely, even across cohorts of the same trial, discordant cut-offs according to tissue or blood sample seemed to significantly affect the reproducibility of the threshold; moreover, other technical issues such as differing turnaround or sample storage time could finally affect TMB values.^54^ Thirdly, the lack of prospective stratification by TMB status in the included RCTs should be considered; withal, mostly owing to the quantity and quality of the available sample, TMB data were not available for all the patients evaluated with differing percentages of TMB-evaluable patients among studies. Furthermore, given that a systematic evaluation of PD-L1 expression levels could not be carried out among the TMB-selected patients, our results should be prospectively confirmed by future RCTs evaluating the efficacy of combined detection of TMB and PD-L1 expression for further implementation of TMB in the clinical practice. Finally, despite the utility of such an approach in selecting patients for immune-based treatments, a cost-effectiveness analysis is eventually needed in the light of the high cost of TMB detection.

## Conclusions

This meta-analysis provided evidence regards the OS benefit in favor of IO agents over CT alone in TMB-selected patients, highlighting the limited utility of CT alone in the TMB-high subgroup. Strikingly, the pooled results of both ORR and PFS in the TMB-low subgroup seemed to favor CT over IO strategies, thus suggesting that high TMB could be predictive for ICI efficacy in the first-line setting. In summary, although more prospective data are warranted, we postulated the hypothesis that monitoring TMB, in addition to the existing PD-L1 expression level, could represent the preferable option for future clinical research in the first-line management of advanced non-oncogene-addicted NSCLC patients.
