# Supplementary material for: The prognostic impact of tumor mutational burden (TMB) in the first-line management of advanced non-oncogene addicted non-small-cell lung cancer (NSCLC): a systematic review and meta-analysis of randomized controlled trials
Source: ESMO Open. 2021 Apr 30;6(3):100124. doi: 10.1016/j.esmoop.2021.100124 (PMC8111593; doi:10.1016/j.esmoop.2021.100124)
Supplement: Supplementary Figure S1 [file mmc1.docx]

**ELECTRONIC SEARCH STRATEGY**

1. We used a search strategy for randomized controlled trials (RCT) in Medline through Pubmed. The same search was then modified for RCT searches in other databases (Scopus, Controlled Cochrane Trial Register - CCTR).

The query was adapted from Robinson & coll: [Robinson KA, Dickersin K. Development of a highly sensitive search strategy for the retrieval of reports of controlled trials using PubMed. International journal of epidemiology 2002;31:150-153].

(("Carcinoma, Non-Small-Cell Lung"[Mesh] OR "NSCLC"[tiab]) AND ("tumor mutational burden"[tiab] OR "TMB"[tiab]) AND "survival"[tiab])

ID Search Hits

#1 NSCLC with Cochrane Library publication date Between Jan 1972 and Sept 2020 8917

#2 tumor mutational burden with Cochrane Library publication date Between Jan 1972 and Sept 2020 145

#3 survival with Cochrane Library publication date Between Jan 1972 and Sept 2020 102568

#4 #1 AND #2 AND #3 with Cochrane Library publication date Between Jan 1972 and Sept 2020 46

2. We used the free text strategy

NSCLC and TMB or Tumor mutational burden and survival

3. We searched meta-analyses for any other published data concerning NSCLC and TMB. The strategy in Pubmed for meta-analysis searching was

Tumor mutational burden [tiab] OR TMB AND "Carcinoma, Non-Small-Cell Lung"[Mesh] OR NSCLC [tiab] AND meta-analysis[pt]

for RCT on-line searches in Clinical Trials registers (www.clinicaltrials.gov)
