# Supplementary material for: The prognostic impact of tumor mutational burden (TMB) in the first-line management of advanced non-oncogene addicted non-small-cell lung cancer (NSCLC): a systematic review and meta-analysis of randomized controlled trials
Source: ESMO Open. 2021 Apr 30;6(3):100124. doi: 10.1016/j.esmoop.2021.100124 (PMC8111593; doi:10.1016/j.esmoop.2021.100124)
Supplement: Supplementary Figure Legends [file mmc3.docx]

**Supplementary Figure S1. Electronic search strategy.**

**Supplementary Figure S2. PRISMA checklist referring to the content of the current systematic review and meta-analysis.**

**Supplementary Figure S3.** Forest plots of (A) RR of ORR, (B) HR of PFS and (C) OS in patients with high TMB assigned to receive single-agent IO (Mono IO) regimens versus CT alone. Abbreviations: CT, platinum-based chemotherapy; HR, hazard ratio; IO, immuno-oncology; ORR, objective response rate; OS, overall survival; PFS, progression-free survival; RR, risk ratio; TMB, tumor mutational burden.

**Supplementary Figure S4.** Forest plots of (A) RR of ORR, (B) HR of PFS and (C) OS in patients with low TMB assigned to receive single-agent IO (Mono IO) regimens versus CT alone. Abbreviations: CT, platinum-based chemotherapy; HR, hazard ratio; IO, immuno-oncology; ORR, objective response rate; OS, overall survival; PFS, progression-free survival; RR, risk ratio; TMB, tumor mutational burden.

**Supplementary Figure S5.** Forest plots of (A) RR of ORR, (B) HR of PFS and (C) OS in patients with high TMB assigned to receive combination IO (Combo IO) regimens versus CT alone. Abbreviations: CT, platinum-based chemotherapy; HR, hazard ratio; IO, immuno-oncology; ORR, objective response rate; OS, overall survival; PFS, progression-free survival; RR, risk ratio; TMB, tumor mutational burden.

**Supplementary Figure S6.** Forest plots of (A) RR of ORR, (B) HR of PFS and (C) OS in patients with low TMB assigned to receive combination IO (Combo IO) regimens versus CT alone. Abbreviations: CT, platinum-based chemotherapy; HR, hazard ratio; IO, immuno-oncology; ORR, objective response rate; OS, overall survival; PFS, progression-free survival; RR, risk ratio; TMB, tumor mutational burden.

**Supplementary Figure S7. Funnel plot assessing the risk of publication bias across the studies.**

**Supplementary Figure S8.** Risk of bias graph: review authors’ judgments about each risk of bias item presented as percentages across all included studies (A); Risk of bias summary (B).
