# Supplementary figures and images for: The prognostic impact of tumor mutational burden (TMB) in the first-line management of advanced non-oncogene addicted non-small-cell lung cancer (NSCLC): a systematic review and meta-analysis of randomized controlled trials
Source: ESMO Open. 2021 Apr 30;6(3):100124. doi: 10.1016/j.esmoop.2021.100124 (PMC8111593; doi:10.1016/j.esmoop.2021.100124)

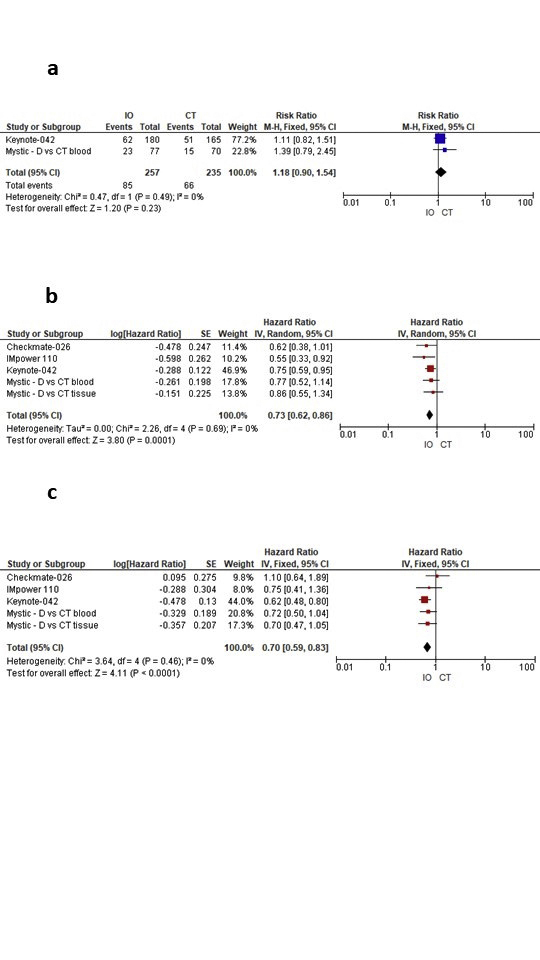

Supplement: Supplementary Figure S3 [file figs1.jpg]

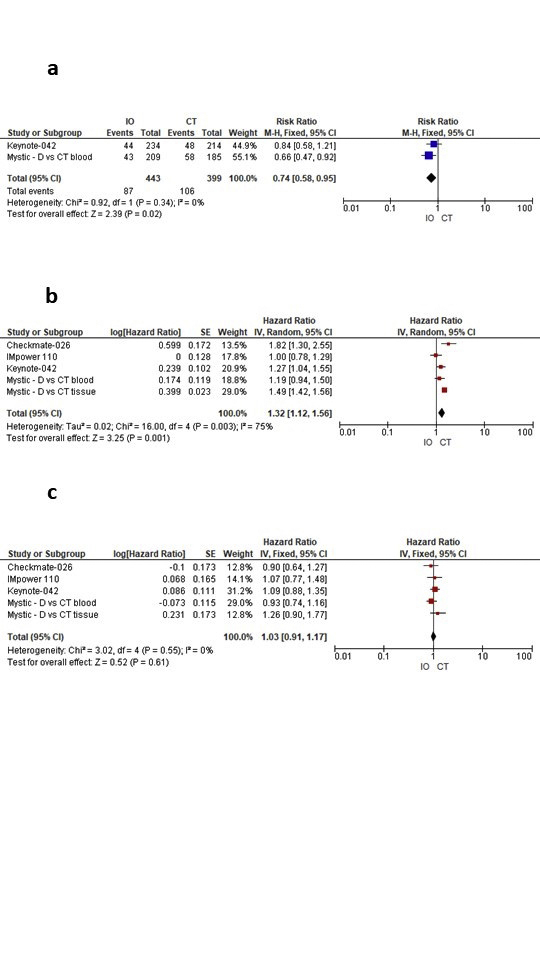

Supplement: Supplementary Figure S4 [file figs2.jpg]

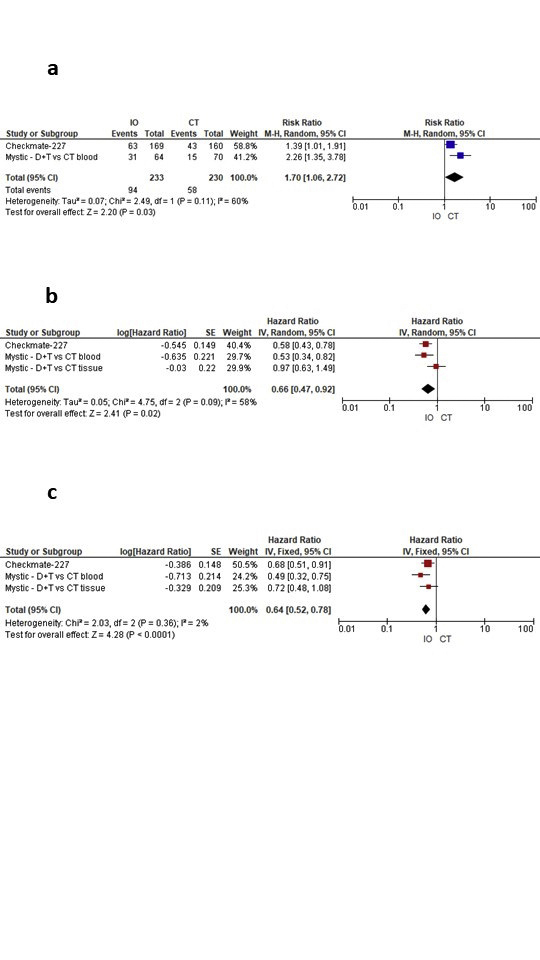

Supplement: Supplementary Figure S5 [file figs3.jpg]

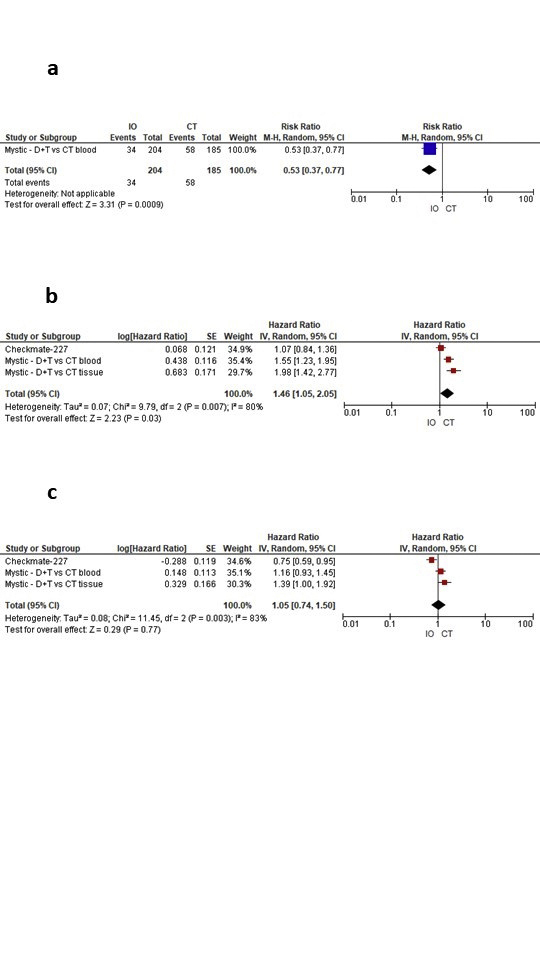

Supplement: Supplementary Figure S6 [file figs4.jpg]

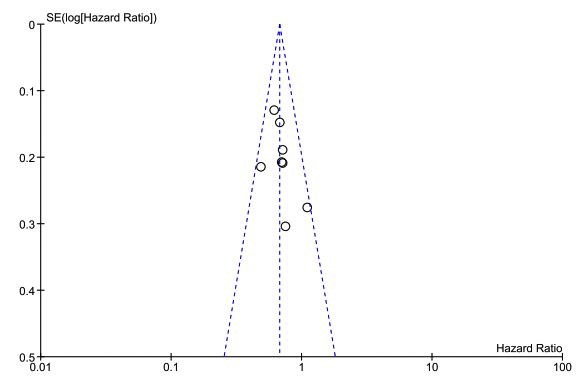

Supplement: Supplementary Figure S7 [file figs5.jpg]

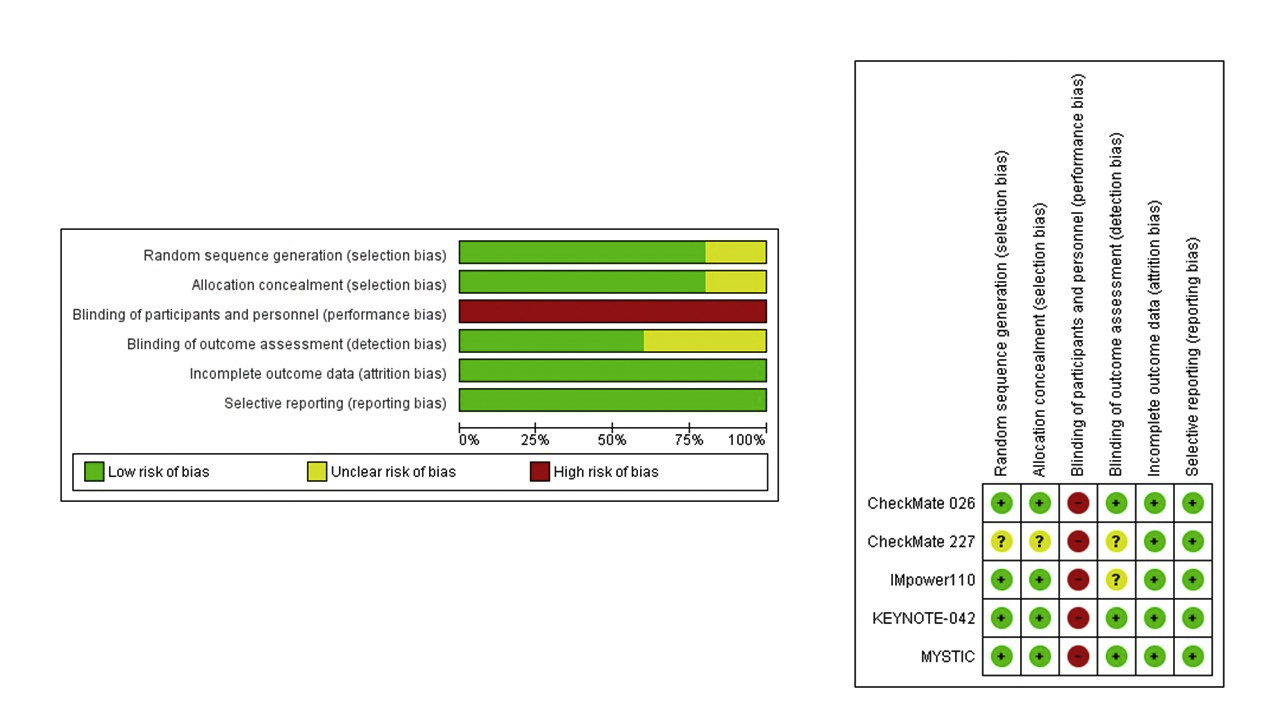

Supplement: Supplementary Figure S8 [file figs6.jpg]
